# Development, propagation and characterization of human somatic cell-derived bronchial organoids as model system for airway diseases – A practical guide

**DOI:** 10.3389/falgy.2026.1811482

**Published:** 2026-06-16

**Authors:** Laura Kühl, Ariana Carvalho, Nicole Löwer, Sarah Miethe, Kim Pauck, Katrin Roth, Nele von Daacke, Pauline Graichen, Eva Müßig, Emma Huy, Anne Mende, Annika Block, Melanie Bachl, Jan Henrik Pflugmacher, Paul Dechert, Daniel P. Potaczek, Atefeh Sadeghi Shermeh, Holger Garn

**Affiliations:** 1 Translational Inflammation Research Division & Core Facility for Single Cell Multiomics, Philipps University of Marburg – Medical Faculty, Member of the German Center for Lung Research (DZL) and the Universities of Giessen and Marburg Lung Center, Marburg, Germany; 2 TissueGnostics GmbH, Vienna, Austria; 3 Cellular Imaging Core Facility, Center for Tumor Biology and Immunology (ZTI), Philipps University of Marburg – Medical Faculty, Marburg, Germany; 4 Center for Infections and Genomics of the Lung (CIGL), Justus Liebig University, Giessen, Germany

**Keywords:** 3D cell culture model system, air-liquid interface (ALI) cultures, airway diseases, airway epithelium, bronchial organoids, human lung organoids, single cell RNA sequencing

## Abstract

Human bronchial organoids represent a highly advanced 3D cell culture model system that reflects complex features of the airway epithelium, including structure, developmental aspects, tissue-specific functions and heterogeneous cell-cell interactions. Thus, they serve as an ideal model to study physiological differentiation and activation processes of the bronchial epithelial cells, as well as pathophysiological mechanisms of lower airway diseases. We have established, refined and validated a series of protocols for the generation, perpetuation and characterization of human bronchial lung organoids based on somatic cells derived from surgical lung tissue samples as well as from primary bronchial epithelial cells which may be obtained from healthy and diseased donors. Such organoids are cultured in an extracellular matrix gel with a serum-free medium containing a precisely adjusted growth factor cocktail. This maintains the balance between the self-renewal and differentiation capacities of the local progenitor cells, allowing the long-term culture of organoids if specific splitting and dilution as well as freezing/thawing procedures are carried out regularly and appropriately. Here we provide these detailed protocols to enable researchers to apply the organoid technology and to generate highly comparable and complementary data. Furthermore, we present several examples for the detailed characterization and analysis of human bronchial organoids, such as gene-level expression analysis, covering single cell RNA sequencing, as well as imaging and metabolic activity-based assays.

## 1 Introduction

Organoids are highly advanced, complex, three-dimensional (3D) cell culture systems composed of various tissue- and organ-specific cell types with self-organizing and self-renewing properties (1). Due to their potential to bridge the methodological gap between simple *in vitro* cell cultures and complex *in vivo* animal model systems, human lung organoids are gaining increasing interest as a disease model system in translational research (2–6).

The human airway epithelium is a complex tissue that plays a fundamental role in the proper functionality of the respiratory system. Impairments on this tissue are involved in a variety of respiratory diseases. In order to understand the structural and functional complexity of human airways in both healthy and diseased conditions, suitable models of human airway biology are necessary. So far, several model systems with different levels of complexity have been in use, ranging from simple cell cultures to sophisticated *in vivo* animal models (7). The simplest experimental approach is the use of immortalized airway epithelial cell lines, such as BEAS-2B or 16HBE14o. The culture of these cells is easy, fast, and reproducible. However, it needs to be considered that they are modified and thus neither accurately represent natural airway cell biology nor the complex structure and function of the healthy or diseased airway epithelium. Alternatively, primary human bronchial epithelial cells (HBECs) can be used. However, their *in vitro* lifespan is limited, and they do not undergo mucociliary differentiation under submerged conditions (8). Therefore, to increase the complexity of *in vitro* cultures, more sophisticated methodologies have already been developed. A step into this direction was the establishment of air-liquid interface (ALI) culture systems, which are generated by polarization of primary HBECs. In this model, the basal side of the culture is in permanent contact with the nutritive medium, while the apical surface of the cell layer is exposed to air, resulting in the development of a pseudostratified mucociliary phenotype (9). Another, even more complex model is based on precision cut lung slices (PCLS), where viable organ specimens are sliced and cultivated *ex vivo* for a certain time (10, 11). This approach has the advantage of essentially containing all cell types present in the human lung in their original 3D architecture. However, PCLS cultures are time-limited and continuous degradation processes may interfere with experimental outcomes. Finally, *in vivo* experiments are performed on animal models, primarily using mice. Although these models represent the highest level of organization, they exhibit a variety of physiological, cellular, and molecular differences to humans. Indeed, the airways of mice and humans differ in many aspects. For example, human bronchioles are lined by a pseudostratified epithelium, whereas the small airways of mice only have a simple epithelium. Therefore, translation of insights obtained from mouse model systems into human airway function needs to be carried out with caution (12). Furthermore, ethical concerns may arise because *in vivo* experiments require the sacrifice of a large number of animals.

Lung organoids offer a comprehensive experimental approach representing the complexity of the entire airway epithelium in a virtually unlimited, continuous 3D cell culture system. They have the potential to reflect complex tissue/organ-specific functions and heterogeneous cell-cell interactions *in vitro* (13). Additionally, unlike in PCLS and ALI cultures, progenitor cell capacities of the basal cells are retained in human lung organoids, enabling their culture over longer periods of time. Distinct media compositions and specific culture protocols are required to achieve this quasi-immortality and promote the differentiation into multiple cell types present in the airway epithelium. This provides the significant advantage of generating a large stock of source material for long-term use through standardized passaging, freezing and thawing procedures.

It is important to note that, depending on the medium components used, lung-derived organoids may resemble either alveolar or bronchial lung structures, or a combination of both (14, 15). Human bronchial organoids are best suited to address scientific questions related to the pathogenesis of diseases that primarily affect the small airways, e.g., bronchial asthma, chronic obstructive pulmonary disease (COPD), viral or bacterial infectious diseases, and cystic fibrosis, as well as their clinical phenotypes and courses (4, 16). In general, bronchial organoids can be derived from induced pluripotent stem cells or from primary somatic epithelial cell preparations obtained from surgical lung samples, bronchial brushes, or from commercially available primary HBECs, provided that they contain a sufficient proportion of progenitors with stem cell-like capacities (17, 18).

Here, we provide detailed protocols for the generation, perpetuation and storage of human somatic cell-derived bronchial organoids. We also present example procedures for their thorough characterization using various molecular and cellular analyses, such as single cell RNA sequencing (scRNA-seq) and reverse transcription quantitative polymerase chain reaction (RT-qPCR), as well as imaging and viability assays.

## 2 Methods

### 2.1 scRNA-seq

Single cell data were generated using the BD Rhapsody system implementing the Single-Cell Capture and cDNA Synthesis with BD Rhapsody Single-Cell Analysis System Protocol (Doc ID: 23-22951(02)) and the BD Rhapsody System mRNA Whole Transcriptome Analysis (WTA) and Sample Tag Library Preparation Protocol (Doc ID: 23-24119(03)). Samples were individually hash-tagged with the BD Flex Single-Cell Multiplexing Kits (Cat. No. 633849-633852) and the respective protocol [Doc ID: 23-24311(01)], combined with a PE-labelled primary antibody against *β*2-microglobulin (Cat. No. 551337; all reagents from BD Biosciences, Heidelberg, Germany) at a 1:5 dilution. The libraries of ∼11,000 captured cells were sequenced on an Illumina Novaseq6000 Sequencer with 57,000 reads/cell at the Genomics and Bioinformatics Platform of the Institute of Lung Health, Justus Liebig University, Giessen, Germany. Raw data were processed using the standard settings on the BD Rhapsody Sequence Analysis Pipeline (Revision: 0) at sevenbridges.com. Processed data was filtered based on the following criteria: mitochondrial percentage <40%, nCount >300, nFeatures between 300 and 10,000 and log10GenesPerUMI > 0.8. One sample was found to dominate the analysis; therefore, it was randomly downsampled to 450 cells, resulting in a total of approximately 7,000 high-quality cells for further analysis.

### 2.2 Stimulation of organoids with poly(I:C)

To test the biological activity of organoids to an external stimulus, organoids cultured for four weeks after last splitting (Supplementary Protocol 3) were stimulated by addition of 5 µg/mL or 20 µg/mL of Poly(I:C) (polyinosinic:polycytidylic acid; Biozol, Hamburg, Germany) to the culture medium for 24 h. Subsequently, organoid cells were isolated and analyzed for inflammatory gene expression by RT-qPCR.

### 2.3 RT-qPCR

Organoid cells were isolated, total RNA was extracted using the RNeasy Mini Kit (Qiagen, Hilden, Germany), and the RNA concentration was determined using a Qubit 4 Fluorometer (Thermo Fisher Scientific, Waltham, MA, USA). Complementary DNA was synthesized using 100 ng of total RNA and the qScript™ Flex cDNA Synthesis Kit (Quantabio, Beverly, MA, USA). qPCR was performed on a Rotor-Gene Q cycler (Qiagen) using QuantiNova SYBR Green Master Mix (Qiagen). The relative expression of the genes of interest was normalized to the housekeeping genes *PGK1* or *RPLP0*. Used primer sequences are provided in Table 1.

**Table 1:** Primer sequences.

Target gene	Forward primer	Reverse primer
*KRT5*	GGGCGAGGAATGCAGACTCA	CACTGCTACCTCCGGCAAGA
*BCAM* (CD239)	CCGGGCTCAGTCTCCGC	GGGTACAGACAAGCGCACCT
*ITGA6*	CTTCTCGCTGGCCATGCAC	TTCTGACGTGGGGTCAGCATC
*SCGB1A1*	TGAAACTCGCTGTCACCCTCA	CGCTGAAAGCTCGGGCAGAT
*MUC5AC*	CAGTGTGAGAAGCACCAGGA	CAGCAGCCGTCCTTGCT
*CEACAM5*	TGCCAGGCGCAGTGATTCAG	GGTGGATTGCTGGAAAGTCCCAT
*FOXJ1*	TACTTCCGCCACGCAGATCC	CCCTTGCCTGGTTCGTCCTT
*TUBA1B*	TCGCCTTCGCCTCCTAATCC	CACTTGGCATCTGGCCATCG
*TNF*	GAGGCCAAGCCCTGGTATG	CGGGCCGATTGATCTCAGC
*IL1B*	AGCTTGGTGATGTCTGGTCC	GCCCAAGGCCACAGGTATT
*CXCL8*	CAGCTCTGTGTGAAGGTGCAGTT	TTTCCTTGGGGTCCAGACAGA
*PGK1*	GTTGACCGAATCACCGACCT	AGCAGCCTTAATCCTCTGGTT
*RPLP0*	GCGTCCTCGTGGAAGTGACAT	GCTTGGAGCCCACATTGTCTG

### 2.4 Live cell imaging of human bronchial organoids

New organoid cultures were set-up following splitting (Supplementary Protocol 3) and maintained in 12-well plates by changing the medium twice a week for two or five weeks. For the latter, the culture was diluted (Supplementary Protocol 4) three weeks after seeding. Live brightfield imaging of the cell culture plates was performed using the inverted microscope THUNDER Imager Cell (Leica Microsystems, Wetzlar, Germany) at 5× and 20× magnification. Furthermore, an organoid culture was maintained to develop a high level of differentiation and Supplementary Video S1 was recorded using the inverted microscope AE31E (Motic, Xiamen, China) at 4× magnification.

### 2.5 Live/dead cell staining

Human bronchial organoids grown for 14 days in TruLive 3D dishes (Luxendo/Bruker, Heidelberg, Germany), as described in Supplementary Protocol 11, were either exposed to 5% dimethyl sulfoxide (DMSO) in 3D Expansion Medium for 24 h or were left untreated. Afterwards, live and dead cells were labelled with 1:1,000 CellTracker Green CMFDA (ThermoFisher Scientific) and 12.5 μM propidium iodide (BD Biosciences), respectively, and nuclei of all cells were stained with 1:1,000 DAPI, according to Supplementary Protocol 11. The samples were imaged using the light sheet microscope TruLive 3D (Luxendo/Bruker) and a 31.25× water immersion objective. Original data were processed by a 2 × 2 binning (X-Y) and converted into Imaris files (Bitplane, Oxford instruments, Abingdon, UK).

### 2.6 Immunofluorescence assay (IFA)

Organoid samples were stained according to Supplementary Protocol 10. The Positive IFA sample was labelled with an anti-E-cadherin polyclonal antibody (1:40; R&D Systems, Minneapolis, MN, USA) and a secondary donkey anti-goat antibody Alexa Fluor 568 (1:1,000; Thermo Fisher Scientific), while the negative control was only incubated with the secondary antibody. Nuclei in both samples were stained with DRAQ5 (1:200; BD Biosciences) and organoids were then embedded in Matrigel (Cultrex Reduced Growth Factor Basement Membrane Extract; R&D Systems). Images and Supplementary Video S2 were captured with the multiphoton microscope FVMPE-RS (Olympus, Hachioji, Tokyo, Japan) and a 25× water immersion objective. The Insight DeepSee parameter was set to 1040 nm.

### 2.7 Metabolic/viability assay using organoid ring cultures

Both the endpoint and kinetic organoid metabolic assays were conducted according to Supplementary Protocol 12. For the endpoint assay, organoids were seeded as ring cultures at three different densities (300, 400 and 500 cells/µL Matrigel). Samples were grown for 10 days and exposed to 20% DMSO in 3D Expansion Medium for the final 72 h, or were left untreated (Medium). For the kinetic assay, organoids were plated at a seeding density of 250 cells/µL Matrigel and grown for two weeks before the metabolic activity of the cells in these organoid cultures started to be repeatedly assessed for two more weeks.

Images of the ring cultures were acquired using the inverted microscope AE31E (Motic) at 4× magnification. The organoid metabolic activity was assessed using the CellTiter-Blue Cell Viability Assay (Promega, Madison, WI, USA). For this, cultures were incubated with the CellTiter-Blue Reagent for four hours at 37 °C with 5% CO_2_ before fluorescence was measured using an Infinite 200 Pro plate reader (Tecan Austria, Grödig, Austria).

### 2.8 Statistics

Statistical analysis of quantitative data was performed using GraphPad Prism (version 10.2.3). Comparisons between two groups were conducted using Welch's *t*-test. Linear correlation analyses were done based on means.

## 3 Results

### 3.1 General procedures for the development and propagation of human bronchial organoids

**Figure 1:**
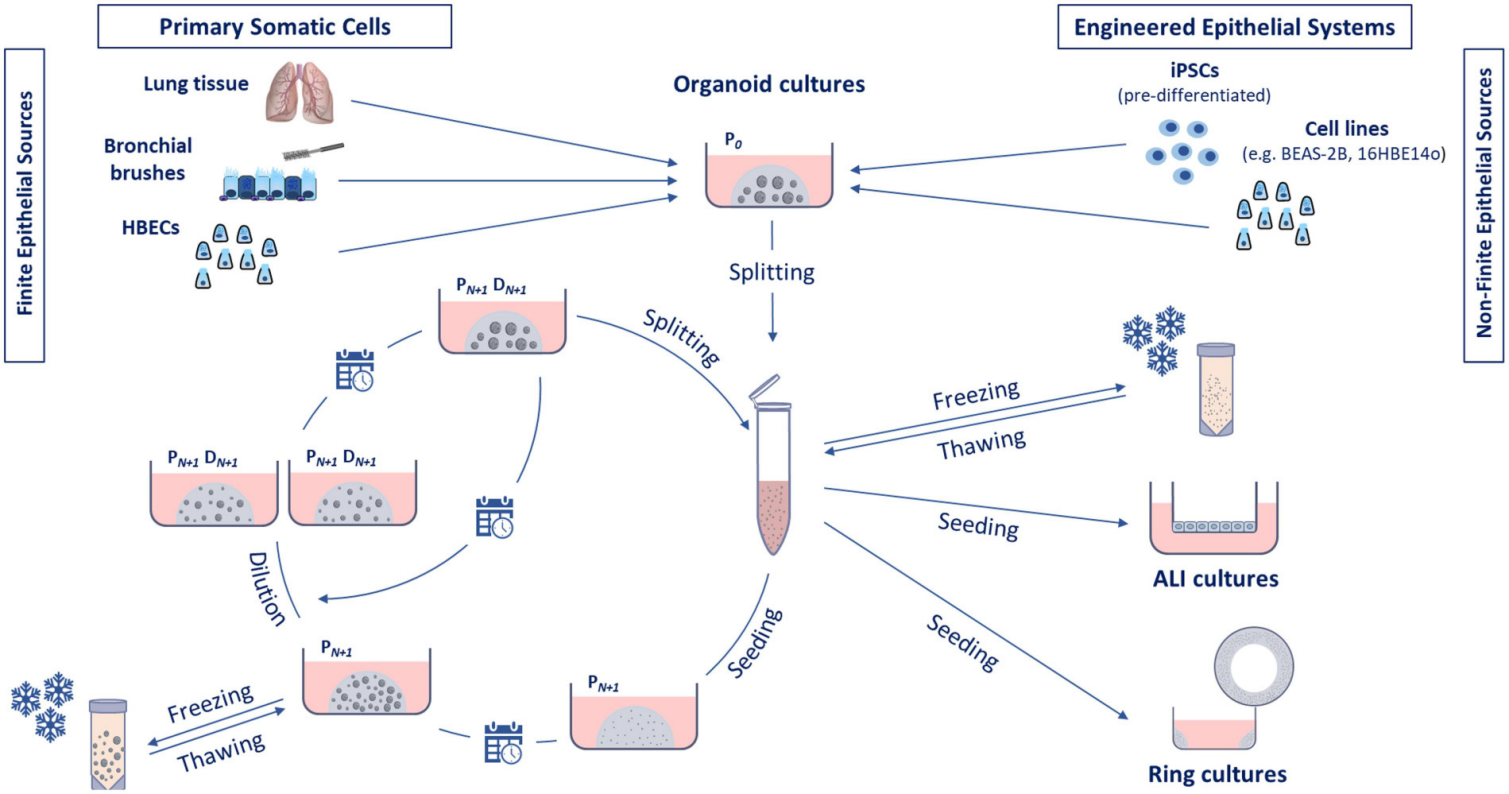
Schematic overview of steps and procedures for the generation and propagation of human bronchial organoids as a continuous culture system. iPSCs, induced pluripotent stem cells; HBECs, human bronchial epithelial cells; P, passage; D, dilution; ALI, air-liquid interface.

Human bronchial organoids representing main structural and functional features of small human airways can be developed from finite epithelial sources, i.e., primary somatic airway cell preparations or from non-finite sources, such as induced pluripotent stem cells (iPSCs), or even cell lines developed from airway cells at early states of differentiation, like BEAS-2B (see Figure 1). Here, we will exclusively focus on methodologies dealing with somatic cell-derived organoids that can be developed using single cell preparations obtained from lung biopsies, surgical explants or bronchial brushings, either directly or from basal (i.e., progenitor) cells isolated thereof.

#### 3.1.1 Establishment of bronchial organoid cultures

To generate organoids from the aforementioned source materials, the initial preparation should be provided as single cell suspension. In case of lung tissue, this can be prepared by initial tissue dissection with a razor blade followed by collagenase digestion and treatment with red blood cell lysis buffer. Single cells are seeded in a drop of a synthetic extracellular matrix, such as Matrigel (i.e., Cultrex Reduced Growth Factor Basement Membrane Extract), which is placed in a well of a cell culture plate. 3D Expansion Medium containing an optimized mixture of growth factors (see Supplementary Protocol 1) is then added to the well, which triggers stem cells with progenitor activity to proliferate and differentiate, thus growing into typical 3D-shaped organoids (for details, see Supplementary Protocol 2). The first small organoids with a lumen can usually be observed after three to five days of culture. Over time, microscopic morphology reveals spherical structures with a visible lumen, mucus production and motile cilia movement inside, corresponding to the apical side of the airway epithelium *in vivo*, as can be seen in Supplementary Video S1.

#### 3.1.2 Propagation of bronchial organoids by splitting and dilution procedures

Long-term propagation and expansion of established organoid cultures over multiple passages can be achieved by a cyclic workflow consisting of two propagation procedures called organoid splitting and organoid dilution (see Figure 1), with the latter being facultative for this process. The term “splitting” describes the process of passaging organoids. In this procedure, the 3D structures of the organoids are disrupted by enzymatic and mechanical processes. The resulting single cell suspension is then seeded in a new drop of Matrigel and, due to the self-renewing and self-organizing capabilities of the present progenitor cells, a new passage of organoids can grow out and develop into new 3D organoid structures (see Supplementary Protocol 3). We recommend performing this procedure 1-2 times at the early stage of organoid development from primary tissue/cells (i.e., before organoids are used for intended experiments) to avoid integration of cells already finally differentiated at the initiation of the organoid cultures. Furthermore, organoid splitting should be applied when analyzing early differentiation mechanisms and the impact of endogenous (e.g., genetic variations, epigenetic alterations due to disease background, inflammatory mediators) or exogenous (e.g., environmental compounds, drugs) factors on these processes is intended.

If organoid cultures need to be expanded because the structures require more space to grow (e.g., due to increased size or number of organoids within the Matrigel drop), but the 3D structure must/should not be destroyed, proceeding with the “dilution” protocol is indicated. In this process, organoids are isolated by dissolving Matrigel at lower temperatures and the resulting suspension is seeded in one or more new drops of Matrigel at a lower organoid density and cultures are continued (see Supplementary Protocol 4). This procedure is recommended for investigating the effects of the above-mentioned impacts on fully differentiated structures with advanced cell composition and established cell-to-cell interactions. For these approaches, it needs to be considered that stimulation occurs from the basal side of the epithelium as this represents the outer layer of the organoids. Dilution of organoids may also be required when bigger organoid structures are to be analyzed or lower densities are advantageous (e.g., for imaging analyses).

The combination of the mentioned methodologies allows the expansion of organoids in both number and size (i.e., number of cells per organoid), enabling multiple experiments to be performed based on identical primary biological source material. It has been demonstrated that gene expression patterns of organoids remain relatively stable over time during culture (13, 19). In addition, for long-term storage, either single cell suspensions obtained following splitting or whole organoids with preserved 3D structure can be cryopreserved in liquid nitrogen and thawed for further use at later time points (see Supplementary Protocols 5-8).

Finally, single cell suspensions obtained by organoid splitting may be used not only to re-establish organoid cultures but also for the initiation of two-dimensional (2D) ALI cultures (see Supplementary Protocol 9) that exhibit very similar properties to those developed from primary human airway cells. In this regard, human bronchial organoids may serve as a continuous supply of human airway cells for ALI culture experiments, with the advantage of conserving the identical biological background for a series of consecutive experiments.

### 3.2 Characterization of human bronchial organoids

#### 3.2.1 Composition and characteristics of human bronchial organoids at different stages of development

The bronchial epithelium consists of different major cell types, including basal cells, secretory cells (such as club cells and goblet cells) and ciliated cells (20, 21). Basal cells are the local progenitor cells of the airway epithelium that may proliferate and differentiate and thus are important for renewal and reparation of the bronchial epithelium. They have the potential to differentiate into all different airway epithelial cells via intermediate basal cell precursors. Different signaling pathways are activated for this purpose, e.g., involvement of the Notch pathway leads to the development of secretory cell lineages, while activation of the c-Myb pathway results in ciliated cell differentiation (22, 23). The underlying activities are regulated by the coordinated action of a number of specific growth factors. Due to a precise mixture of such factors added to the 3D Expansion Medium (for details see Supplementary Protocol 1), which provide balanced signals for proliferation and differentiation, basal cells present in the initial lung cell suspension are enabled to initiate organoid formation. Further differentiation processes, as well as the intermediate cell states, are also observed during organoid development and growth (see below).

#### 3.2.2 Single cell analysis of bronchial organoids

**Figure 2:**
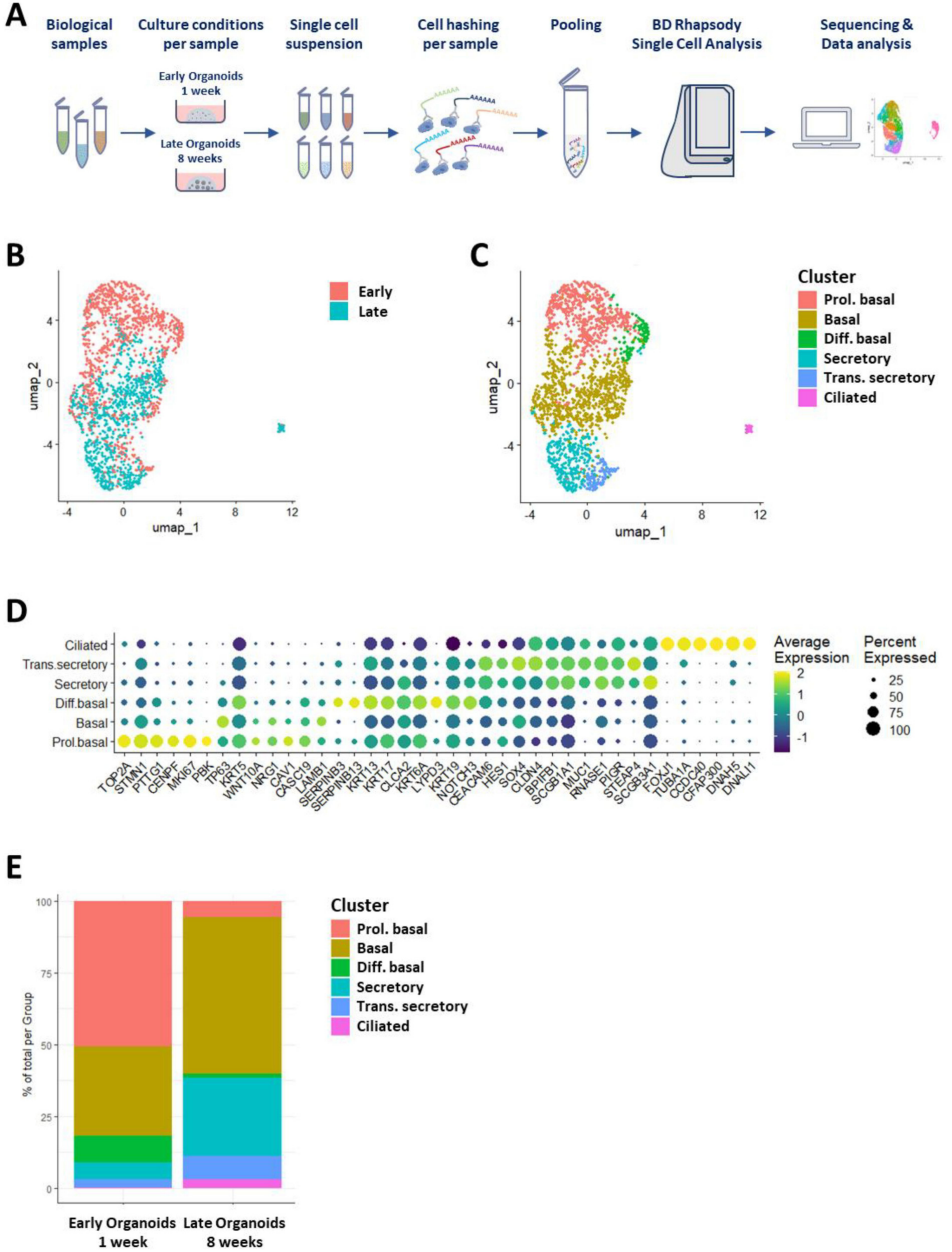
Comparative single cell RNA sequencing-based characterization of organoids at two time points of culture. **(A)** Schematic overview of the experimental workflow. Bronchial lung organoids derived from three independent biological samples were cultured for one week (early organoids) or eight weeks (late organoids) after last splitting. Organoids were dissociated into single cell suspensions which were individually hash-tagged, pooled, and processed for scRNA-seq using the BD Rhapsody platform. **(B)** UMAP visualization of single cells colored by culture condition. **(C)** UMAP representation of main epithelial cell populations annotated based on canonical marker gene expression. **(D)** Dot plot showing scaled, averaged expression of selected marker genes across annotated epithelial cell populations. **(E)** Stacked bar plot showing the relative proportion of cell clusters identified in **(C)** in organoid cells from the two culture conditions. scRNA-seq, single cell RNA sequencing; Prol. basal, proliferating basal; Diff. basal, differentiating basal; Trans. secretory, transitional secretory.

scRNA-seq was employed to obtain an unbiased, high-resolution view of the cellular composition of bronchial organoids, enabling assessment of epithelial heterogeneity and differentiation states. To demonstrate kinetics and robustness of the organoid culture system, organoids from three independent biological replicates were maintained for two different periods of time, i.e., one week or eight weeks following last splitting, here called “early” and “late” organoids, respectively (Figure 2A). The results of a Uniform Manifold Approximation and Projection (UMAP) analysis of single cells derived from early and late culture conditions of bronchial organoids, based on scRNA-seq data, are shown in Figure 2B. Coloring by culture conditions reveals a separation between cells derived from early and late organoids. Early organoid cells are predominantly localized in the upper region of the UMAP, and late organoid cells are enriched in the lower part, indicating that distinct transcriptional states are associated with culture duration. Unbiased Phenograph-based clustering resulted in six main epithelial cell clusters (Figure 2C), which were annotated based on established reference annotations reported in previous publications (24, 25). Cell identities were inferred based on the relative enrichment and specificity of canonical marker genes across clusters, including proliferating basal cells (*TOP2A, STMN1, MKI67*), basal cells (*TP63, KRT5*), differentiating basal cells (*KRT5, KRT13, KRT17, KRT19*), secretory cells (*SCGB1A1, SCGB3A1, RNASE1*), transitional secretory cells (*SOX4, CLDN4, SCGB1A1, STEAP4, CEACAM6*), and ciliated cells (*FOXJ1, TUBA1A, DNAH5, CCDC40*) (Figure 2D). As shown in Figure 2E, organoids at one week of culture consist predominantly of basal cells at different levels of differentiation with about 50% of all cells being proliferating basal cells. This indicates the high (proliferative) activity of progenitor cells at early time points of organoid development. In contrast, more differentiated cells such as secretory, transitional secretory, and some ciliated cells represented a markedly larger fraction of total cells in late organoids at eight weeks of culture, indicating a progressive differentiation process in such organoids over longer time periods. Alveolar type 1 and type 2 cells could not be detected at any time point of analyses, which can be attributed to the specific composition of the culture medium and contained growth factors favoring the differentiation of progenitor cells into bronchial cell types and preventing differentiation into alveolar cells.

#### 3.2.3 RT-qPCR characterization and functional validation of bronchial organoids

**Figure 3:**
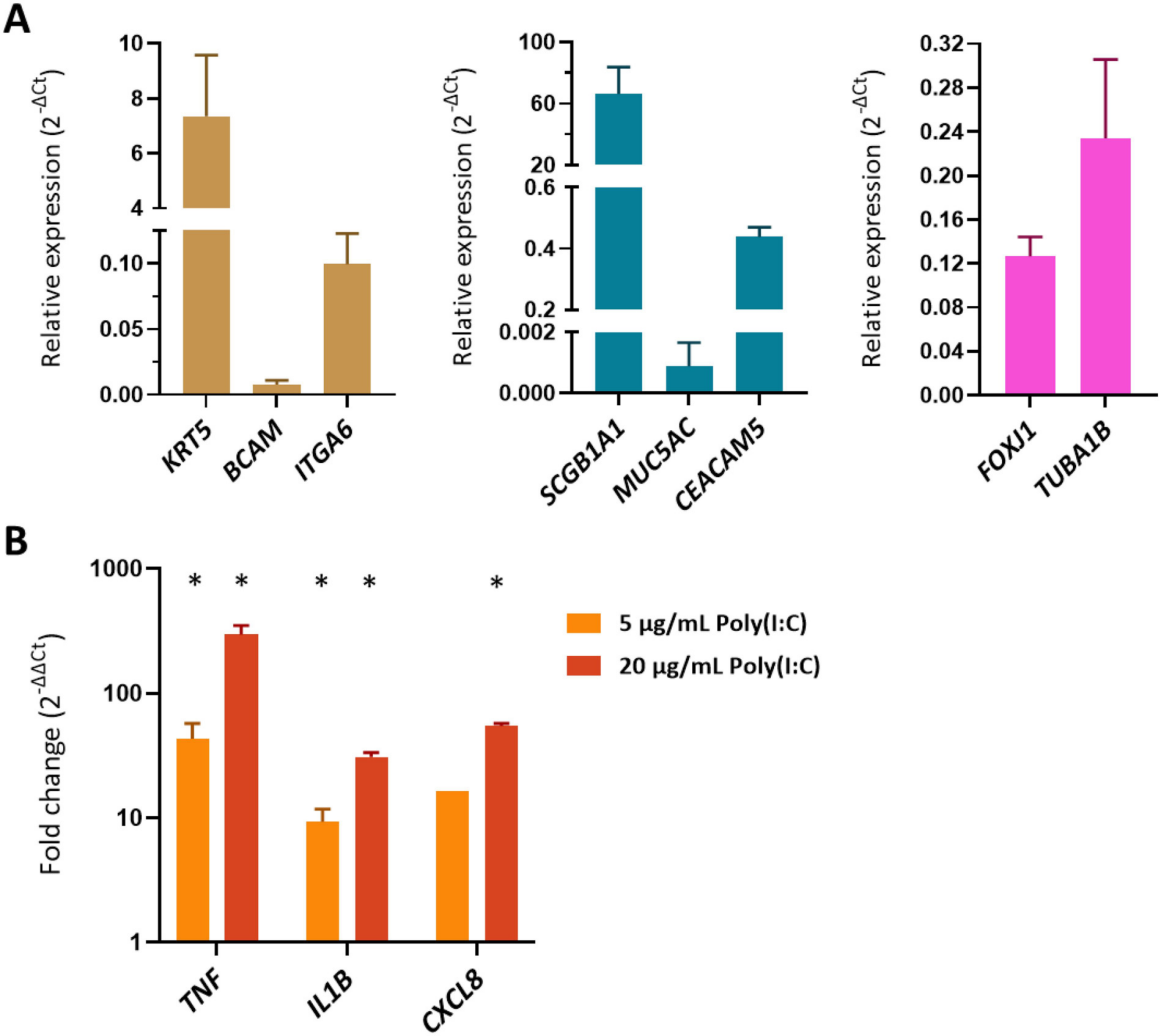
Human bronchial organoid characterization by gene expression analysis via RT-qPCR. **(A)** Baseline expression of selected marker genes for main epithelial cell subtypes assessed in a bronchial organoid culture at 12 weeks following last splitting. The three panels show marker gene expression profiles for basal (left), secretory (middle), and ciliated (right) cells. Data are shown as relative expression normalized to the housekeeping gene *PGK1* (2^−*ΔCt*^). **(B)** Expression of pro-inflammatory cytokine genes analyzed in an organoid culture four weeks after the last splitting, following 24 h stimulation with two different concentrations of Poly(I:C), to assess the functional responsiveness of bronchial organoids. Relative expression levels of *TNF*, *IL1B*, and *CXCL8* are shown in comparison to the mock control, after normalization to the housekeeping gene *RPLP0*. Shown are means + standard deviation of *n* = 2 replicates. Statistical testing was performed using delta Ct values and significant differences to the untreated controls are indicated by (*) for *p*-values < 0.05.

Transcript-level characterization of organoids can also be performed using simpler and more widely used methods, such as RT-qPCR. Here we show an example using a panel of primers identifying established epithelial markers to indicate the presence of basal cells (*KRT5*, *BCAM*, and *ITGA6*), secretory cells (*SCGB1A1, MUC5AC*, and *CEACAM5*), and ciliated cells (*FOXJ1* and *TUBA1B*) in a human bronchial organoid culture at 12 weeks following seeding (see Figure 3A) (7, 20, 26, 27). The high expression of the basal and secretory lineage markers *KRT5* and *SCGB1A1* confirms the dominant presence of these cell types while the rather low expression of *FOXJ1* and *TUBA1B* hints to a lower abundance of ciliated cells even at this later stage of organoid development. However, RT-qPCR analysis of lineage markers provides a rather qualitative characterization of epithelial identity. It does not allow for the direct quantification of epithelial subtype proportions due to gene-specific differences in expression levels (as for example indicated by the lower expression of *BCAM* or *MUC5AC*), differential expression in cell subtypes (e.g., resident secretory cells vs. activation-induced goblet cells), and/or concomitant expression of lineage-specific markers, e.g., in transitional cell states.

Furthermore, to demonstrate the functional responsiveness of organoids, the expression of pro-inflammatory cytokine genes was investigated following stimulation with the Toll-like receptor-3 agonist Poly(I:C), a synthetic analog of viral dsRNA (28). For this purpose, bronchial organoid cultures at four weeks following seeding were treated with Poly(I:C) at two different concentrations (5 µg/mL and 20 µg/mL) for 24 h. Subsequent RT-qPCR analysis revealed a dose-dependent up-regulation of the selected genes (*TNF*, *IL1B*, and *CXCL8*) in response to Poly(I:C) treatment compared to untreated control conditions (Figure 3B).

### 3.3 2D and 3D imaging of bronchial organoids

**Figure 4:**
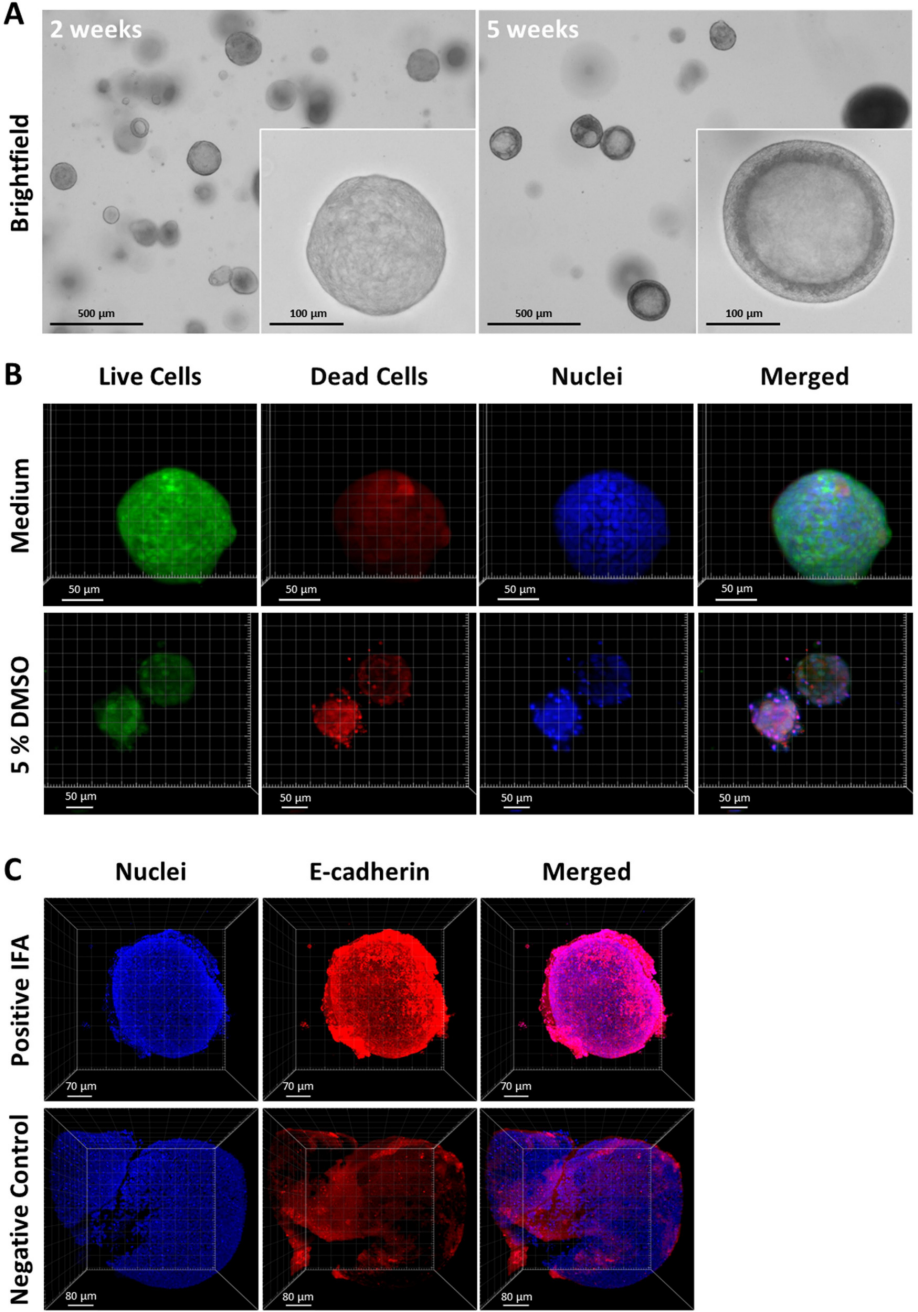
Examples for different imaging analyses of human bronchial organoids via three different techniques. **(A)** Live cell imaging using brightfield microscopy of cultures derived from the same organoid line at two (left) and five (right) weeks after seeding. The scale bars correspond to 500 µm and 100 µm (insets). **(B)** Live/dead cell staining imaged with light-sheet microscopy on two fixed samples, a medium control (upper panel) and a sample exposed to 5% DMSO for 24 h (lower panel). Live cells are marked with CellTracker Green CMFDA, dead cells with propidium iodide, and nuclei with DAPI. Right panels show merged images of the three fluorescence channels; scale bars correspond to 50 µm. **(C)** Fixed organoids positively stained for E-cadherin expression (upper panel) vs. negative control with primary antibody omitted (lower panel). Nuclei were stained with DRAQ5 and samples were imaged using multiphoton microscopy. Merged images of both fluorescence channels are shown in the right panel, scale bars correspond to 70 µm and 80 µm in Positive IFA and Negative Control, respectively. IFA, immunofluorescence assay; DMSO, dimethyl sulfoxide.

In addition to the described molecular biology methods, imaging analyses may further contribute to organoid characterization. The dilution protocol (Supplementary Protocol 4), which may be repeatedly executed, enables the culture of human bronchial organoids for several weeks, which is particularly advantageous for achieving increased organoid complexity with higher levels of differentiation into all major airway epithelial cell types. This coincides with the development of the typical 3D-appearance of bronchial organoids, as shown in Figure 4A, demonstrating organoids with a well-defined lumen after five weeks in culture (right panel) in contrast to organoids that have been in culture for only two weeks (left panel).

In addition to analyzing organoid morphology via brightfield microscopy, specific antigens (e.g., markers of different cell populations) can be visualized and analyzed using IFA in association with state-of-the-art microscopy techniques. This includes confocal laser scanning, multiphoton and light-sheet microscopy, which generate high-resolution images and 3D digital reconstructions. Organoids can be stained with histological dyes or labelled antibodies to further investigate their viability or architecture. As example of an organoid viability assay that can be conducted to visualize and count dead and live cells in 3D, Figure 4B shows human bronchial organoids cultured under standard conditions (upper panel) or in presence of 5% DMSO for 24 h as a toxic impact (lower panel). Using two dyes, CellTracker Green CMFDA and propidium iodide, live and dead cells were marked, respectively. Following fixation with 4% paraformaldehyde, the nuclei were stained with DAPI. The organoids under the toxic impact show disrupted structures with several cells having popped out. This negative impact on organoid viability is further supported by the observation that the majority of cells are labelled with the dead cell marker. By contrast, dead cells are almost entirely absent from the medium sample, which instead shows a stronger signal from the live cell marker. Additionally, this sample preserves the spherical shape with intact outer cell layer, which is associated with healthy appearance of human bronchial organoids.

Furthermore, immunofluorescence staining enables a more detailed investigation of the 3D structure, cellular composition and molecular characteristics of organoid samples, which might be of special interest when using them as disease model systems or drug screening platforms. As an example, Figure 4C shows human bronchial lung organoids labelled through indirect immunofluorescence (upper panel) in comparison to an unstained negative control (lower panel). Staining was performed with antibodies against E-cadherin, a marker of epithelial cells. The positive staining signal in this sample is clearly stronger than the background fluorescence observed in the negative control, which derives solely from Matrigel embedding after staining. Supplementary Video S2 represents the Positive IFA sample scanned throughout Z levels.

Importantly, when using technologies other than multiphoton microscopy, an additional clearing step may become required prior to imaging in order to clearly identify positive signals from the inner layers of the organoid 3D structures (29). In the human bronchial organoid model, clearing with an alkaline solution containing a combination of 2,2′-thiodiethanol, DMSO, D-sorbitol, and Tris proved to be the most effective approach (not shown) (30). This step should be conducted at the end of the staining protocol (see Supplementary Protocol 10).

### 3.4 Metabolic/viability assay using organoid ring cultures

**Figure 5:**
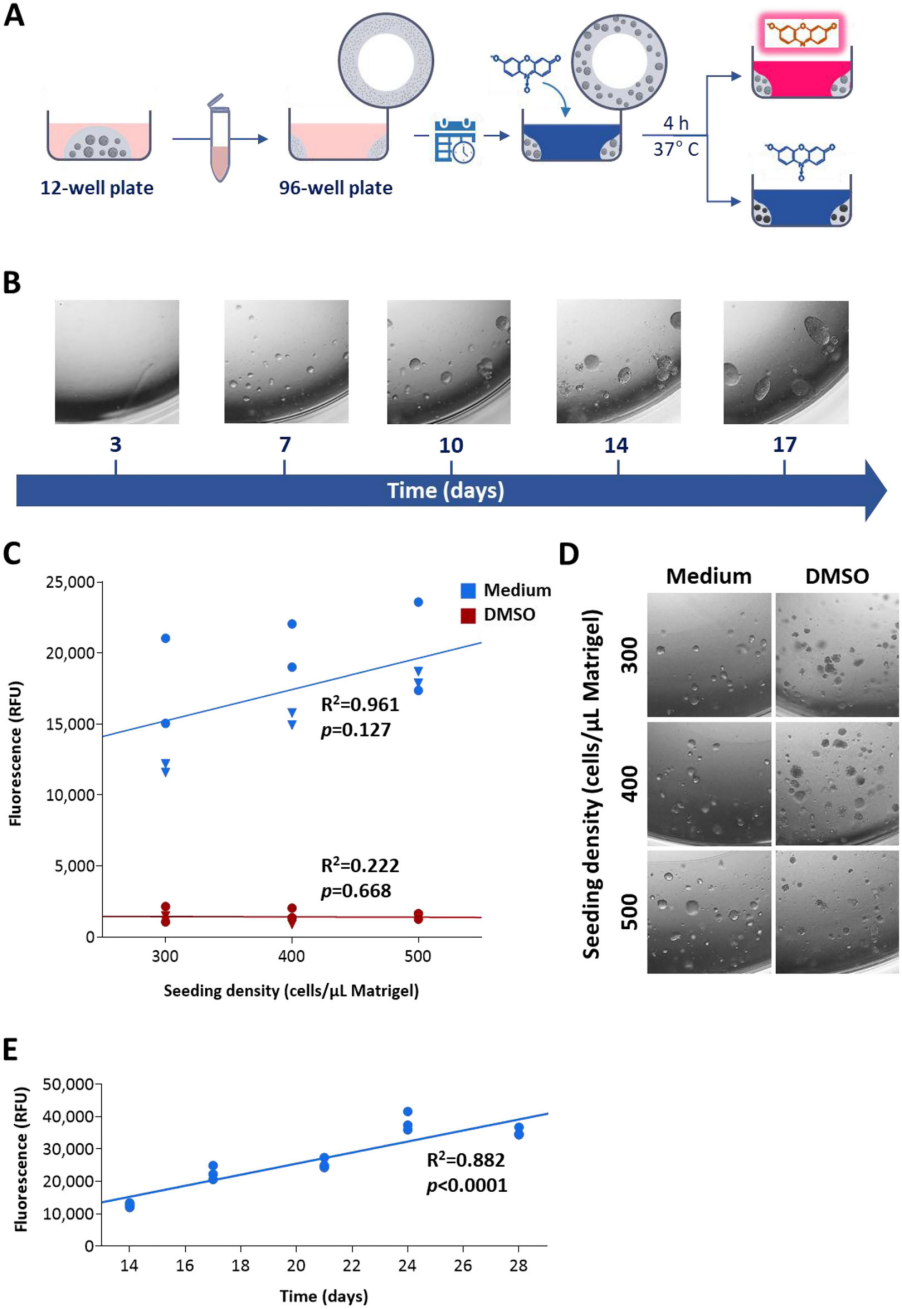
Metabolic activity/cell viability assessed in human bronchial organoid ring cultures as either an endpoint or a kinetic assay. **(A)** Schematic overview of the method. Split organoid cells were seeded in 96-well plates as a ring and cultured for certain periods of time. To assess metabolic activity, CellTiter-Blue reagent (resazurin) is added to the wells and incubated for four hours during which it is reduced to resorufin by metabolic cell activity. The resulting fluorescence signal is then quantified. **(B)** Brightfield images demonstrating growth of the organoids seeded as ring cultures over time. **(C)** Metabolic activity and **(D)** representative images of organoid cultures seeded at different cell densities and kept for 10 days in culture under standard conditions (Medium) or exposed to 20% DMSO for the last 72 h. Symbols represent two organoid lines, each analyzed in duplicate. **(E)** Kinetic analysis of the metabolic activity of organoid cultures, starting from the second week after seeding (250 cells/µL Matrigel). Organoid cultures were kept under standard conditions in triplicate. Metabolic activity was analyzed repeatedly over a period of 2 weeks. Lines in **(C)** and **(E)** represent linear regression, and coefficients of determination (R^2^) and *p*-values are provided. DMSO, dimethyl sulfoxide.

All analytical procedures described above represent endpoint assays requiring the ultimate termination of the organoid cultures. Furthermore, standard procedures of organoid propagation in Matrigel drops prevent the conduct of simple microplate-based screening assays that would be helpful, e.g., for toxicity testing of pharmacological compounds. Here, we provide a methodology that overcomes these limitations by applying the widely used CellTiter-Blue Cell Viability Assay to 3D organoid cultures for endpoint and kinetic analyses. This allows high-throughput analyses of multiple conditions and automated measurements as known from standard cell culture approaches (31). For this methodology, organoids are seeded and grown as ring cultures in 96-well plates (see Supplementary Protocol 12), in which the growth of the organoids over time can also be visualized by microscopy (see Figures 5A,B). Test compounds may be applied to the culture medium at any timepoint of interest and the CellTiter-Blue reagent is added to monitor the metabolic activity/viability of the cultures. For kinetic assays, this procedure may be performed repeatedly.

To exemplify an endpoint analysis, two different organoid lines were seeded at three different densities (300, 400, and 500 cells/µL Matrigel) and cultured for 10 days with or without 20% DMSO for the last 72 h before CellTiter-Blue reagent was added and fluorescence measurements were conducted. Expectedly, higher cell densities showed a clear trend to result in higher fluorescence signals which dropped drastically following DMSO treatment (Figure 5C). Microscopic imaging of the Matrigel rings corroborated these results (Figure 5D).

Finally, a kinetic assay was performed analyzing one organoid line in triplicates with a first measurement at 14 days after seeding of 250 cells/µL Matrigel. CellTiter-Blue reagent incubation and fluorescence measurements started at this time point and were repeated twice a week for the next two weeks. Fluorescence signals gradually increased over time due to the continuous growth of the organoid structures with a significant correlation between these parameters (Figure 5E).

## 4 Discussion

To fulfill its main function, i.e., gas exchange to supply the body with oxygen, the respiratory system is a complex organ composed of a variety of different cell types specialized in taking on specific tasks. Recent investigations using single cell sequencing technologies have shown that, in contrast to the distal alveoli, this is specifically true for the small airways, which are characterized by a high degree of heterogeneity in terms of cell types and their activation states (20, 21). This indicates that cells of the small airways perform a variety of different and specialized functions, altogether aimed at protecting the alveolar region from harmful environmental influences. Therefore, disturbances in this carefully balanced system may result in serious and chronic diseases like bronchial asthma or COPD (32, 33).

Appropriate models that reflect the structural and functional complexity of airway structures are required to analyze the dynamics of epithelial differentiation and activation processes under physiological and pathophysiological conditions in detail. Recent developments in biomedical research have shown that bronchial organoids in combination with state-of-the-art analytical methods are ideally suited to take on this role (4, 6, 34). Therefore, it is advised to develop and apply standardized protocols for generating, perpetuating and characterizing these complex structures. Following this concept, we here provide a set of well-established and practically proven protocols for the handling of bronchial organoids with the idea to enable the scientific community to generate highly comparable and complementary data.

We specifically focused on the generation of somatic cell-derived organoids from lung tissue source material. Such samples can either be obtained through biopsies taken from potential cancer patients—ideally from those where the existence of a tumor could be excluded—or by surgical removal of lung tumors. Tumor-surrounding, unaffected healthy tissue can be used to generate organoids (35); however, a potential impact of the existing comorbidity needs to be considered. On the other hand, these protocols can easily be adopted for primary cells obtained by bronchial brushings, the preparation of which has been described elsewhere (18). Given the higher ratio of epithelial to non-epithelial cells in bronchial brushings, a lower seeding number is sufficient compared to organoid generation from human lung tissue samples (8, 36, 37). This approach improves the feasibility of organoid generation from patients with obstructive airway diseases, such as bronchial asthma, COPD, bronchiolitis or cystic fibrosis, and healthy subjects, thus facilitating the direct investigation of disease-associated perturbations in epithelial differentiation and activation processes.

The protocols presented here are robust but, at the same time, comparatively short, since others often include harvesting solutions that require longer incubation times (38) or coating of the plates with Matrigel followed by an overnight incubation period (39). Further refinement of the initial steps is possible by starting procedures based on the isolation of progenitor basal cells, e.g., by flow cytometry sorting based on CD271/nerve growth factor receptor expression (40, 41).

As the described organoid approach is based on primary source material from human individuals, there is certainly a variability between organoid lines derived from different donors. While reproducibility and efficiency in the general development of organoids from different individuals is quite high (> 90% to our experience), growth and differentiation kinetics may differ. Thus, as for other studies based on primary human cell material, it is advised to perform experiments using at least three biological replicates (i.e., organoid lines from three different donors) to take the interindividual variability into account. Heterogeneity may be even higher when cells derived from patients suffering from airway diseases are used to develop organoids for subsequent mechanistic studies, due to differences in disease phenotype, progression or severity, treatment history, and comorbidities. Accordingly, we recommend conducting such studies based on source material from well-defined patients with similar characteristics regarding the above-mentioned confounding factors.

Like any model system, organoids present advantages and limitations. The absence of other cell types and the continuous regenerative capacity allow the focused investigation of epithelial differentiation processes and regeneration or repair mechanisms over long periods, with the prospect of future personalized medicine applications. However, the absence of additional cell types, such as immune and stromal cells, limits their physiological complexity. ALI cultures also exhibit epithelial differentiation, but, unlike organoids, they have a limited lifespan and exhibit a virtually synchronized aging process due to the gradual loss of progenitor cell activity (42). PCLS, in turn, preserve native lung architecture including vasculature, stromal and immune cells. Further, they may be subjected to mechanical forces mimicking breathing activity. However, their *in vitro* lifetime is even shorter, with distinct kinetics in viability and activity of different cell types (7, 11).

On the other hand, the complexity of organoid systems may be further enhanced by co-culture with certain types or mixtures of homologous immune cells, fibroblasts or tumor cells (43, 44). These modifications offer the opportunity to study complex cell-cell interactions of the aforementioned cell types with complex epithelial structures outside of a living organism. Moreover, while having the great advantage of generating a human-based complex *in vitro* system, it should be noted that the protocols provided here can also be used to develop organoids from mouse lungs. This offers the possibility to investigate mechanisms of epithelial differentiation in long-lived structures both from wildtype and genetically modified mice, which paves the way for significantly reducing the number of mice used in *in vivo* experiments.

Organoids are considered to reflect features of the normal or pathological state of their origin as they may maintain intrinsic gene expression programs during their cultivation for extended time periods, which has recently been demonstrated in intestinal and bronchial organoids (13, 18, 45). Taking this into account, bronchial organoids can be used not only to better understand the underlying pathophysiological mechanisms, but also to analyze and optimize personalized therapy strategies for patients suffering from diverse lung disorders, even beyond the above-mentioned obstructive airway diseases (16, 46, 47). That includes lung tumors, e.g., by testing the potential efficacy of alternative anti-tumor therapies, including immunotherapy approaches at an individualized level *in vitro* (48). Similarly, organoids generated from subjects suffering from genetically determined diseases may support the development of personalized treatment strategies for these patients. As an example, correction of mutated alleles of monogenic disorders using novel CRISPR/Cas9-mediated approaches has been successfully demonstrated to result in organoids with restored functionality (49). Therefore, somatic cell-derived organoids may specifically serve as an ideal model system for investigating underlying pathophysiological mechanisms of the mentioned diseases and for developing stratified prevention and treatment concepts for such disorders, e.g., as a part of personalized medicine approaches.

Taken together, human bronchial organoids represent a highly advanced structural and functional *in vitro* model of the human airway epithelium. Due to their very high level of organization and complexity, they reproduce the *in vivo* situation of human airways much better than most other cell culture technologies, under both healthy and diseased conditions. Hence, when applied in combination with the huge repertoire of available and applicable analytical methods, this sophisticated 3D model can ideally address a multiplicity of different scientific questions.

## Data Availability

The single cell sequencing data for this study have been deposited in the European Nucleotide Archive (ENA) at EMBL-EBI under accession number PRJEB112513 (https://www.ebi.ac.uk/ena/browser/view/PRJEB112513). All other original data are available on reasonable request from the corresponding author.

## Supplementary material

The Supplementary Material for this article including detailed experimental protocols can be found online at: https://www.frontiersin.org/articles/10.3389/falgy.2026.1811482/full#supplementary-material

Supplementary Video S1 Cilia movement within a human bronchial organoid.

Supplementary Video S2 Z stacks movie through the Positive IFA sample shown in Figure 4.
