# Supplementary material for: Development, propagation and characterization of human somatic cell-derived bronchial organoids as model system for airway diseases – A practical guide
Source: Front Allergy. 2026 Jun 16;7:1811482. doi: 10.3389/falgy.2026.1811482 (PMC13314865; doi:10.3389/falgy.2026.1811482)
Supplement: Supplementary file 3 [file Datasheet1.pdf]

Online Supplement

Development, propagation and characterization of human somatic cell-derived bronchial organoids as model system for airway diseases – A practical guide

Laura Kühl[*], Ariana Carvalho[*], Nicole Löwer, Sarah Miethe, Kim Pauck, Katrin Roth, Nele von Daacke, Pauline Graichen, Eva Müßig, Emma Huy, Anne Mende, Annika Block, Melanie Bachl, Jan Henrik Pflugmacher, Paul Dechert, Daniel P. Potaczek, Atefeh Sadeghi Shermeh[#], Holger Garn[#§]

[*]Equal contribution first authors

[#]Equal contribution last authors

[§]Corresponding author

Corresponding author address:

Prof. Dr. Holger Garn

Philipps University of Marburg, Medical Faculty,

Translational Inflammation Research Division & Core Facility for Single Cell Multiomics

Center for Tumor Biology and Immunology (ZTI)

Hans-Meerwein-Str. 3

D - 35043 Marburg, Germany

Phone: #49 6421 2866040

E-Mail: garn@staff.uni-marburg.de

PROTOCOLS

As outlined and shown in the main manuscript, human bronchial organoids can be generated from a variety of sources. Here, we describe routine approaches and provide detailed protocols for the generation and propagation of somatic cell-derived human bronchial organoids such as splitting, dilution, freezing and thawing, according to which such organoids can be maintained for several years. Furthermore, we describe a number of assays and procedures that can be performed using this complex model to evaluate the impact of physiological or environmental factors on activation and differentiation processes of airway epithelial cells. Throughout these protocols, the following general recommendations should be taken into account:

- If not otherwise indicated, all procedures must be performed under sterile conditions.
- The serum-free 3D Expansion Medium is crucial for organoid maintenance, as it contains growth factors essential for the proliferation and differentiation of airway progenitor cells, known as basal cells. As some of its components are light-sensitive, it must always be kept away from light.
- Prior to being added to culture plates, the 3D Expansion Medium must be pre-warmed to 37 °C. Medium should be changed twice a week to maintain established organoid cultures, always by carefully aspirating the used medium from the plate and replacing it with fresh, pre-warmed medium.
- Before use, Matrigel must be thawed either on ice or overnight at 4 °C. Additionally, plates intended for seeding can be pre-warmed at 37 °C to facilitate plating.
- Air bubbles in Matrigel must be avoided, since these may affect the stability of the drops, organoid growth and imaging. If air bubbles are present in the Matrigel, placing it at 4 °C will allow the bubbles to disappear. Also, during seeding, the tube containing the cell/Matrigel mixture must be kept on ice for as long as possible to prevent its solidification prior to plating and the formation of bubbles by pipetting.
- For handling of suspensions with formed organoids (e.g. during dilution, freezing/thawing or staining), the ends of the pipette tips used should be cut off to prevent disruption of the 3D structures by shear forces.

Protocol 1. Preparation of 3D Expansion Medium

1.1. Prepare DMEM/F12 Comfort Medium with supplements (hereafter referred to as Basic Medium) by pipetting the exact amounts of each component listed in Table 1.

Table 1 *- Basic Medium mix*

Component	Volume
DMEM/F12 Comfort Medium	485 mL
GlutaMAX Supplement (100X)	5 mL
Hepes Solution 1 M	5 mL
Penicillin-Streptomycin Solution 100X	5 mL
Primocin 50 mg/mL	500 µL

NOTE: The remaining 15 mL of DMEM/F12 Comfort Medium can be stored at 4 °C to be used in the preparation of freezing medium (see Supplement Protocol 5).

1.2. Prepare the 3D Expansion Medium by pipetting the exact amounts of all components together according to Table 2. Store finished 3D Expansion Medium at 4 °C in the dark.

NOTES:

- *All components must be thawed on ice, except for A 83-01 and SB 202190, which should be thawed at room temperature (RT) and protected from light.*
- *The N-acetylcysteine solution must be freshly prepared. All components must be sterile, which can be achieved by filtering solutions through a 0.2 µm syringe filter.*

Table 2 *- 3D Expansion Medium mix (50 mL)*

Component	Volume	Final Concentration
Basic Medium	43.2 mL	
Noggin	50 µL	100 ng/mL
R-Spondin-1	5 mL	500 ng/mL
NC-27 Supplement	1 mL	1x
Y-27632 dihydrochloride	25 µL	5 µM
FGF-7	12.5 µL	25 ng/mL
FGF-10	50 µL	100 ng/mL
A 83-01	25 µL	500 nM

SB 202190	25 µL	500 nM
Nicotinamide (500 mM)	500 µL	5 mM
N-Acetylcystein (500 mM)	125 µL	1.25 mM

Protocol 2. Generation of bronchial organoids from human lung tissue samples

This section describes how to initially generate bronchial organoids from surgical lung tissue samples or lung biopsies.

Preparation steps: Pre-warm the 3D Expansion Medium and thaw the Matrigel on ice, or overnight at 4 °C. The cell culture plate (12-well or 24-well) used for seeding can be pre-warmed at 37 °C for easier plating.

2.1. Use a scalpel to dissect and mince the lung tissue in a petri dish until the pieces are small enough to fit through a pipette tip.

2.2. Wash the petri dish with 1 mL of Basic Medium and transfer all of the dissected lung pieces to a 15 mL tube. Repeat the washing step to yield a total volume of 5 mL and add 5 mL of 2 mg/mL collagenase for further tissue dissociation.

2.3. Incubate by rotating the tube for 2 hours in a humidified atmosphere at 37 °C.

2.4. Shear the cell solution first with a 10 mL and then again with a 5 mL pipette. Afterwards, filter it consecutively through a 100 µm filter and 30 µm filter into a new 15 mL tube.

2.5. Add 200 µL FCS to the 10 mL cell suspension, and centrifuge for 5 minutes at 400 g and 4 °C.

2.6. Discard the supernatant and resuspend the cell pellet in 1 mL of red blood cell lysis buffer and incubate for 4 minutes at RT.

2.7. Add 9 mL of Basic Medium to stop lysis, filter cells through a 30 µm filter and centrifuge for 5 minutes at 400 g and 4 °C.

2.8. Discard the supernatant, resuspend the cell pellet in 5 mL sterile PBS and count cells.

2.9. Prepare a vial with the appropriate volume of the cell suspension to be seeded, considering that each 40 µL Matrigel drop should contain 2.5×10^5 cells. Repeat centrifugation (5 minutes, 400 g, 4 °C) and then discard the supernatant.

RECOMMENDATION: After centrifugation, a small volume of up to 40 µL of the supernatant can be left in the vial, which will make the next step easier.

2.10. Resuspend the cell pellet in an appropriate amount of ice cold liquid Matrigel (40 µL

per drop). Place one drop per well if using a 24-well plate or up to three drops per well in a 12-well plate.

2.11. Incubate the plate in a humidified atmosphere for up to 30 minutes at 37 °C with 5 % CO_2, until the Matrigel has solidified.

2.12. Add 500 µL or 1 mL pre-warmed 3D Expansion Medium per well in the 24- or 12-well plate, respectively. Carefully pipette the medium onto the side wall of the well to avoid breaking down the Matrigel drops.

2.13. Keep organoid cultures in a humidified incubator at 37 °C with 5 % CO_2. The medium should be changed twice a week, always by carefully aspirating the used medium from the plate and replacing it with fresh, pre-warmed medium.

Protocols for the propagation of human bronchial organoids

This section describes the splitting and dilution procedures, which serve distinct but complementary purposes in organoid culture and should be selected based on the experimental aim. Organoid splitting, i.e. disrupting the 3D structures into a single cell suspension and subsequently regenerating new complex organoids, is necessary to maintain and expand the culture when the organoids have reached a certain size and/or exhibit signs of stress. Splitting is also used to reset cultures when the investigation of initial differentiation processes is of interest. In contrast, dilution aims to distribute intact organoids while preserving their 3D structure across additional Matrigel drops, e.g. when drops become too crowded, thereby maintaining healthy growth of intact organoids for longer periods of time.

For both procedures, particular attention should be paid to the chosen splitting or dilution factor, as excessively low organoid or cell densities may impair optimal organoid formation and continued growth. Depending on the number and size of organoids in the original culture, dilution factors of 1:2 to 1:3 are generally recommended. This decision should be guided by the remaining space available for further growth and the growth rate of the organoids, which may vary depending on tissue origin or underlying disease status. When optimal conditions are uncertain, it is advisable to begin with a lower factor, as both splitting and dilution procedures can be repeated if necessary.

If, at any point during cultivation, a Matrigel drop breaks, it can be directly transferred into a 15 mL tube, and either the splitting or dilution protocol can be performed as appropriate. For experimental setups requiring different culture volumes or an increased number of wells, organoids may be cultured in larger or smaller well plates by scaling the amount of Matrigel drops and 3D Expansion Medium accordingly. Conversely, if large organoids are desired, they may be maintained within a single Matrigel drop with sufficient space for several weeks, provided that the medium is changed regularly.

Protocol 3. Splitting of human bronchial organoids

Preparation steps: Same as for Protocol 2.

3.1. Discard the medium from the plate by carefully pipetting to prevent aspiration of the Matrigel drops.

3.2. Add 1 mL of ice-cold sterile PBS to each well. Gently scrape the bottom of the well with the pipette tip to detach the Matrigel drops and pipette up and down to completely liquefy the Matrigel drops.

3.3. Transfer the whole suspension into a 15 mL tube and repeat the washing step until all the organoids have been recovered from the well. It is recommended that at least two drops from the same source are pooled in one 15 mL tube.

3.4. Fill the 15 mL tube with ice-cold sterile PBS up to 10 mL and wash the organoids by gently pipetting up and down. Centrifuge the tube for 5 minutes at 400 g and 4 °C.

3.5. Carefully discard the supernatant using a pipette tip, resuspend the pellet in 1 mL TrypLE Express and incubate at 37 °C for 7 minutes.

3.6. Shear the organoid suspension by resuspending it with a handmade, flamed glass Pasteur pipette, and immediately inactivate the enzyme activity by adding 5 mL ice-cold Basic Medium.

NOTE: To achieve a single cell suspension, it is advisable to use handmade flamed glass Pasteur pipettes. These are prepared by flaming the tip of a standard Pasteur pipette over a Bunsen burner. The smaller opening of the pipette allows for better cell separation and lower cell loss during subsequent filtration.

3.7. Filter the suspension through a 30 µm filter into a new 15 mL tube. Press the remaining cell solution through the filter using the plunger top of a 1 mL syringe and then wash the filter twice with 2 mL ice-cold Basic Medium, always with pressing using the plunger.

NOTE: Pressing the remaining cells through the filter with the plunger is necessary to minimize cell loss during filtration. However, it must be done carefully to avoid damaging the filter.

3.8. Centrifuge for 5 minutes at 400 g and 4 °C, then discard supernatant completely using a pipette tip. Remove as much supernatant as possible without disrupting the cell pellet.

3.9. Resuspend the cell pellet in 1 mL Basic Medium, then count the cells.

3.10. Repeat centrifugation (5 minutes, 400 g, 4 °C) of an appropriate volume of the cell suspension containing $2x10^4$ cells per drop. Discard the supernatant and resuspend the pellet in ice-cold liquid Matrigel (40 µL per drop).

RECOMMENDATION: After centrifugation, a small volume of up to 40 µL of the supernatant can be left and used to resuspend the cell pellet.

3.11. Place one 40 µL drop per well if using a 24-well plate, or up to three drops per well in a 12-well plate. Incubate the plate in a humidified atmosphere at 37 °C with 5 % CO_2 for up to 30 minutes, until the Matrigel drops have solidified.

3.12. Add 500 µL or 1 mL pre-warmed 3D Expansion Medium per well in the 24- or 12-well plate, respectively, by carefully pipetting the medium onto the side wall of the well to avoid breaking down the drops.

3.13. Keep organoid cultures in a humidified incubator at 37 °C with 5 % CO_2.

Protocol 4. Dilution of human bronchial organoids

Preparation steps: Same as for Protocol 2. Moreover, cut off the ends of the pipette tips used in this protocol to avoid disrupting the organoid structures by shear forces.

4.1. Aspirate the medium from the wells to be diluted and add 1 mL ice-cold sterile PBS to each well to liquefy the Matrigel. Gently scrape the bottom of the well with the pipette tip to detach the Matrigel drops and pipette up and down to completely dissolve the Matrigel without damaging the organoids.

4.2. Transfer the suspension into a 15 mL tube and wash the well again with ice-cold PBS until all the organoids have been recovered from the well. Several drops containing organoids from the same source can be pooled in one 15 mL tube.

4.3. Fill the 15 mL tube with ice-cold sterile PBS up to 10 mL and centrifuge for 5 minutes at 400 g and 4 °C.

4.4. Discard the supernatant using a pipette tip without disrupting the pellet. Wash the organoid pellet again by adding 10 mL sterile, ice-cold PBS and gently mixing with a pipette.

4.5. Repeat centrifugation (5 minutes, 400 g, 4 °C) and then discard the supernatant by pipetting.

RECOMMENDATION: After centrifugation, a small volume of up to 40 µL of the supernatant can be left in the vial and used to resuspend the pellet.

4.6. Resuspend the organoid pellet in an appropriate volume of ice-cold liquid Matrigel (40 µL per drop), depending on the desired dilution ratio.

4.7. Place one 40 µL drop per well if using a 24-well plate, or up to three drops per well in a 12-well plate. Incubate the plate in a humidified atmosphere at 37 °C with 5 % CO_2 for up to 30 minutes, until the Matrigel has solidified.

4.8. Add 500 µL or 1 mL pre-warmed 3D Expansion Medium per well in the 24- or 12-well plate, respectively, by carefully pipetting it onto the side wall of the well to avoid breaking down the drops.

4.9. Keep organoid cultures in a humidified incubator at 37 °C with 5 % CO_2.

Protocols for freezing and thawing of human bronchial organoids

Like cell lines or primary cells, single cell suspensions derived from split organoid cultures can be kept frozen in liquid nitrogen for long-term storage. Similarly, also intact organoids can be deep-frozen in liquid nitrogen for long-term storage while preserving their 3D structure. For both procedures, gradual, stepwise cooling is important to maximize post-thawing cell viability. This can be achieved using different freezing approaches, such as use of a freezing container with isopropanol pre-cooled at 4 °C or other validated stepwise cooling methods.

It is important to consider that the initial organoid growth rate is usually lower directly after thawing single cell suspensions as compared to when seeding split organoids. Therefore, the seeding density must be considerably higher than in Protocol 3. *Splitting of human bronchial organoids*, as described below. Furthermore, when thawing whole organoids, they may appear slightly damaged immediately after thawing, but they are expected to regain their normal, spherical morphology after several days in culture.

Protocol 5. Freezing of split single cell suspensions from human bronchial lung organoids

Preparation steps: Prepare freezing medium by adding 10 % DMSO and 10 % FCS to DMEM/F12 Comfort Medium (see Step 1.1.) and keep it on ice.

5.1. Follow steps 3.1. to 3.9. of Protocol 3. *Splitting of human bronchial organoids*.

5.2. Centrifuge an appropriate volume of the cell suspension, containing 2.5×10^5 to 2×10^6 cells per cryovial, for 5 minutes at 400 g and 4 °C.

5.3. Discard the supernatant using a pipette tip, resuspend the pellet in ice-cold freezing medium, and immediately transfer 1 mL to each cryovial. Work quickly from now on.

5.4. Perform the gradual, stepwise cooling method. If using a freezing container filled with pre-cooled 500 mL isopropanol, immediately place the cryovials inside the freezing container and transfer it to a -80 °C freezer for 24 hours. It is recommended that cryovials are then transferred to a liquid nitrogen tank for long-term storage.

Protocol 6. Thawing of single cell suspensions to regenerate human bronchial organoids

Preparation steps: Same as for Protocol 2. Furthermore, fill a 15 mL tube with 5 mL Basic Medium and place it on ice until use.

6.1. Thaw the frozen cells at 37 °C by gently swinging the cryovial in a water bath until only a small amount of ice remains. Immediately proceed to the next step.

6.2. Transfer all the suspension into the 15 mL tube pre-filled with 5 mL ice-cold Basic Medium.

6.3. Follow steps 3.8. to 3.12. of Protocol 3. *Splitting of human bronchial organoids* but increase the seeding density mentioned in step 3.10. to $3\text{-}4 \times 10^4$ cells per drop.

Protocol 7. Freezing of whole human bronchial organoids

Preparation steps: Same as for Protocol 5. Additionally, cut off the end of the pipette tip used in this protocol to avoid disrupting the organoid structures by shear forces.

7.1. Follow steps 4.1. to 4.5. of Protocol 4. *Dilution of human bronchial organoids*.

7.2. Carefully resuspend the pellet in an appropriate volume of ice-cold freezing medium (1 mL per cryovial) without damaging the organoid structures.

7.3. Transfer the organoid suspension into a labelled cryovial, and quickly perform the gradual, stepwise cooling method. If using a freezing container filled with pre-cooled 500 mL isopropanol, immediately place the cryovials inside the freezing container and transfer it to a -80 °C freezer for 24 hours. It is recommended that cryovials are then transferred to a liquid nitrogen tank for long-term storage.

Protocol 8. Thawing of whole human bronchial organoids

Preparation steps: Same as for Protocol 6. Moreover, cut off the end of the pipette tip used in this protocol to avoid disrupting the organoid structures by shear forces.

8.1. Thaw the frozen organoids at 37 °C by gently swinging the cryovial in a water bath until only a small amount of ice remains. Immediately proceed to the next step.

8.2. Transfer all the suspension into the 15 mL tube pre-filled with 5 mL ice-cold Basic Medium.

8.3. Follow steps 4.5. to 4.9. of Protocol 4. *Dilution of human bronchial organoids*. The volume of Matrigel to be used should be decided based on the organoid count or confluency per Matrigel drop at the time of freezing. This should allow the organoids to be re-seeded with enough space to recover over several days or weeks before further procedures are carried out.

Protocol 9. Air-liquid interface cultures using human bronchial organoid-derived single cell suspensions

Air-liquid interface (ALI) cultures differ from organoids in important aspects that should be considered when choosing between these methods. On one hand, ALI cultures derived from split organoids also have the ability to differentiate, particularly upon exposure to air on their apical side (air lift). However, unlike organoid cultures, this process is time-limited, with continuous loss of the progenitor cells through differentiation, which is usually complete after six to eight weeks. On the other hand, since ALI cultures grow as 2D layers in an insert, they are an appropriate primary cell-derived model for studying certain influences from both the apical and basal sides of the epithelial layer. It is recommended that this protocol is performed using single cell suspensions derived from the splitting of organoid cultures, rather than from freshly thawed cells.

Preparation steps: The day before seeding, prepare the 24-well plate(s) to be used by adding a 0.4 µm pore size insert to each well. At the seeding day, pre-warm the Airway Medium and the 3D Expansion Medium.

9.1 Prepare a filter-sterile working solution of 0.01 % collagen in high-purity distilled water and pipette 150 µL of this solution into the apical chamber of each insert. Incubate the plate overnight at 37 °C.

9.2 The following day, aspirate the remaining collagen volume from the inserts and leave them to dry with the lid open on the laminar flow hood for at least 20 minutes.

9.3 Follow Protocol 3. *Splitting of human bronchial lung organoids*, from step 3.1. to 3.9. In the meantime, perform the next step as soon as the inserts have dried.

9.4 Prepare a mixture of 3D Expansion Medium (75 %) and Airway Medium (25 %), considering that 700 µL is required per well. Then add 600 µL to the basal chamber and 50 µL to the apical chamber of each well and allow the collagen to soak for some minutes before seeding.

9.5 Count the cells in the suspension obtained from splitting the organoids, and prepare a vial with the desired volume, considering that about $5x10^4$ cells must be seeded per insert.

9.6 Centrifuge for 5 minutes at 400 g and 4 °C, then discard the supernatant.

9.7 Resuspend the pellet in the medium mixture (50 µL per well) and seed the cells by carefully pipetting 50 µL of the cell suspension into the apical chamber of each coated and soaked insert.

9.8 Gently swirl the plate to mix and spread the cells throughout the whole area of the chamber before incubating at 37 °C and 5 % CO_2.

9.9 The medium must be changed twice a week, by completely aspirating the medium from the plate without disturbing the cells on the insert and then adding 100 µL of fresh, pre-warmed medium to the apical chamber and 600 µL to basal chamber in each well. During the first two weeks, the medium must become successively more enriched with Airway Medium than the initial mixture used on the seeding day, as follows:

- Medium change 1: 50 % 3D Expansion Medium, 50 % Airway Medium
- Medium change 2: 25 % 3D Expansion Medium, 75 % Airway Medium
- Medium change 3 onwards: 100 % Airway Medium

9.10 When the cell layer reaches approximately 95 % confluency, perform air lift, i.e., the medium in the apical chamber should be discarded and the culture maintained by changing the medium in the basal chamber only. Importantly, when mucus production is observed, the apical side should be carefully washed with 150 µL pre-warmed, sterile PBS at each medium change.

Protocol 10. Immunofluorescence assay with whole human bronchial organoids

This protocol describes how to label antigens via indirect immunofluorescence for *in situ* detection using fluorescence microscopy, while preserving the 3D structure of the organoid cultures. This protocol can be performed under non-sterile conditions, but strict safety precautions must be taken during the initial steps, as PFA is a hazardous material.

Preparation steps: Pre-cool PBS, Fixative solution (4 % PFA) and the centrifuge to 4 °C.

10.1. Aspirate the medium from the plate and add an appropriate volume of the ice-cold Fixative solution, in order to fully cover all the culture. Shake plate for 15 minutes at 4 °C. Afterwards, swirl the plate by hand to check if all the Matrigel has dissolved. If the organoids are free from the Matrigel, proceed to step 10.2.; if not, incubate for additional 10 minutes at 4 °C.

RECOMMENDATION: From now on, it is recommended to work with low-retention tubes and pipette tips with cut-off ends for optimal handling and minimal loss of the fixed organoids. Pre-rinsing the tubes and tips with 6 % BSA is an effective alternative to commercially available low-retention materials.

10.2. Transfer the organoids into a 1.5 mL microcentrifuge tube by pipetting up and down until they are well separated and any remaining Matrigel has been dissolved. Wash the well with PBS to recover all the organoids into the tube.

10.3. Centrifuge for 5 minutes at 800 g and 4 °C. Afterwards, carefully discard the supernatant, without disrupting the pellet.

10.4. Wash by resuspending the pellet in 500 µL PBS and incubate for 10 minutes at RT while rotating.

10.5. Repeat step 10.3. and then resuspend organoid pellet in 500 µL NH_4Cl (50 mM in PBS) and incubate for 10 minutes at RT while rotating.

10.6. Repeat steps 10.3. and 10.4., followed by 10.3. again.

10.7. Resuspend pellet in 500 µL Permeabilization buffer (0.5 % Triton X-100 in PBS) and incubate for 75 minutes at RT while rotating.

10.8. Repeat step 10.3. and then resuspend the pellet in 500 µL Blocking buffer (5 % serum + 0.5 % Triton X-100 in PBS). Incubate for 1 hour at RT while rotating. Meanwhile,

prepare the required Primary staining solution(s) with the primary antibody(s) and respective Blocking Buffer and centrifuge the solution(s) for 10 minutes at 12,000 g at 4 °C.

NOTE: The serum used in the Blocking Buffer must match the source of the secondary antibody to be used.

10.9. Repeat step 10.3. and resuspend the pellet in 500 µL Primary staining solution. Incubate at 4 °C for 12-16 hours while rotating.

10.10. Repeat steps 10.3. and 10.4., followed by 10.3. again. Meanwhile, prepare the required Secondary staining solution(s) with the secondary antibody(s) and respective Blocking Buffer and centrifuge for 10 minutes at 12,000 g at 4 °C.

10.11. Resuspend pellets in 500 µL Secondary staining solution and incubate for 3 hours at RT protected from light while rotating.

10.12. Repeat steps 10.3. and 10.4., followed by 10.3. again.

10.13. Resuspend pellets in 350 µL of nuclei staining solution (1:200 DRAQ5 or 1:1,000 DAPI in PBS) and incubate for 20 minutes at RT protected from direct light while rotating.

10.14. Repeat steps 10.3. and 10.4., followed by 10.3. again. Add PBS again.

10.15. The organoids can now be imaged in PBS, or, if they should be fixed in place, resuspended in 0.5 % low melting point agarose and placed in plates with appropriate optical properties for high-resolution microscopy.

NOTE: Depending on several factors, such as the microscope used, the organoid size, the cell density and the level of mucus production, an additional step of clearing may be required for optimal 3D imaging of the stained samples.

Protocol 11. Live/dead cell staining of whole human bronchial organoids

This protocol can be performed to assess organoid viability *in situ* using light sheet microscopy.

Preparation steps: On seeding day, pre-warm the 3D Expansion Medium and thaw the Matrigel on ice, or overnight at 4 °C. This microscope requires V-shaped chambers (TruLive 3D Dishes) and holders for organoid culture. On the final day, pre-warm 4 % PFA.

11.1. Follow Protocol 3. *Splitting of human bronchial organoids*, from step 3.1. to 3.9.

11.2. Transfer the required volume of the cell suspension to a new vial, considering each V-shaped chamber will be seeded with $7.5x10^3$ cells.

11.3. Centrifuge for 5 minutes at 400 g and 4 °C. Then, discard the supernatant and resuspend the pellet in an appropriate volume of ice-cold Matrigel, considering that the required volume per chamber is 15 µL.

RECOMMENDATION: After centrifugation, a small volume of the supernatant can be left and used to resuspend the pellet.

11.4. Plate drops of 15 µL in the center of each chamber and incubate in a humidified atmosphere at 37 °C and 5 % CO_2 for up to 30 minutes, until the Matrigel has solidified.

11.5. Add 300 µL pre-warmed 3D Expansion Medium or testing solution to each chamber and maintain the culture for as long as desired.

11.6. On the final day of exposure to the testing conditions, replace the medium in each chamber with 250 µL staining solution consisting of CellTracker Green (1:1,000) and Propidium iodide (12.5 µM) in sterile PBS. Incubate for 45 minutes at 37 °C and 5 % CO_2.

NOTE: From this step onwards, samples must be protected from light to preserve optimal fluorescence for microscopy.

11.7. Replace the staining solution with 200 µL pre-warmed 4 % PFA per chamber and incubate for 30 minutes at 37 °C.

11.8. Wash three times with PBS for 5 minutes each at RT.

11.9. Permeabilize cells by incubating with 0.1 % Triton X-100 in PBS (permeabilization

buffer) for 30 minutes at RT.

11.10. Replace solutions in each chamber with 150 µL DAPI (1:1,000 in permeabilization buffer) and incubate for 30 minutes at RT.

11.11. Repeat step 11.8. At the final washing step, do not discard the PBS and image the samples as soon as possible with a light sheet microscope.

Protocol 12. Metabolic/viability assay using organoid ring cultures

This protocol outlines a method for evaluating the effects of treatments and/or environmental factors on the metabolic activity/viability of epithelial cells in 3D organoid structures. This provides a flexible method that can be used as either an endpoint or kinetic assay. The protocol was developed using the CellTiter-Blue Cell Viability Assay kit.

Preparation steps: Same as for Protocol 2 using 96-well plate(s).

12.1. Plan the layout of the plate to be seeded, considering every condition should be seeded at least in duplicate and include the relevant controls, which may include:
- (untreated) organoid control (cultured with 3D Expansion Medium only)
- toxic control (e.g., organoids cultured in 20 % DMSO, or pure, sterile water)
- background control (Matrigel without cells)

12.2. Follow Protocol 3. *Splitting of human bronchial organoids*, from step 3.1. to 3.9.

12.3. Prepare a vial with the required volume of the cell suspension, considering that each well should be cultured with 5 to $7x10^3$ cells.

NOTE: A pre-test is recommended to identify the optimal seeding density, as this can vary significantly depending on cell source and time cells will be kept in culture during the assay.

12.4. Pellet the cells by centrifugation for 5 minutes at 400 g and 4 °C. Then, discard the supernatant and resuspend the pellet with an appropriate volume of ice-cold Matrigel, considering that the required volume per well is 20 µL.

RECOMMENDATION: After centrifugation, a small volume of up to 40 µL of the supernatant can be left and used to resuspend the pellet.

12.5. Plate the cell suspension in the pre-warmed plate as a 20 µL ring per well by swirling the pipette tip around the bottom of each well and leaving the center free. For background measurement, plate Matrigel rings without any cells in the respective background control wells.

12.6. Incubate the plate in a humidified atmosphere at 37 °C and 5 % CO_2 for up to 30 minutes, until the Matrigel rings have solidified. Then, add 100 µL pre-warmed 3D

Expansion Medium to each well by gently pipetting onto the side wall of the well.

12.7. Organoid cultures must be grown for at least a week before the medium is replaced with that of the experimental conditions to be tested. To do this, aspirate the medium by pipetting from the center of the well without disturbing the Matrigel ring, then add 100 µL of the corresponding testing solution by gently pipetting onto the side wall of the well.

12.8. For the measurement of metabolic activity, add 20 µL of CellTiter-Blue Reagent to the medium present in each well. Carefully swirl the plate for around 10 seconds to ensure thorough mixing, then incubate for 4 hours at 37 °C with 5 % CO_2. Afterwards, measure fluorescence using a microplate reader with an excitation wavelength set to 579 nm and an emission wavelength of 584 nm.

NOTE: The CellTiter-Blue Reagent aliquots and plate should be protected from light at all times.

12.9. If the culture is to be kept growing for reassessment at later timepoints (kinetic assay), aspirate the solution of medium and CellTiter-Blue from the plate without disturbing the Matrigel rings, gently wash the wells twice with 250 µL pre-warmed PBS, and add again 100 µL of the corresponding testing solution to the side wall of each well.

12.10. Repeat steps 12.8. and 12.9. at any timepoints of interest.

MATERIALS AND EQUIPMENT

Table 3 *- Reagents*

Item	Company<br>Address	Cat. No.
**A 83-01**	Tocris Biosciences<br>Bristol, UK	2939
**Airway Epithelial Cell Growth Medium**	Promocell<br>Heidelberg, Germany	C-21060
**Ammonium chloride (NH4CL)**	ACROS Organics<br>Geel, Belgium	396400010
**Bovin Serum Albumin Powder**	Anprotec<br>Bruckberg, Germany	AC-AF-0006
**CellTiter-Blue Cell Viability Assay**	Promega Corporation<br>Madison, WI, USA	G8081
**CellTracker Green CMFDA**	Thermo Fisher Scientific<br>Grand Island, NY, USA	C7025
**Collagen**	Sigma-Aldrich<br>St. Louis, MO, USA	C9791
**Collagenase**	Sigma-Aldrich	C0130
**Cultrex Reduced Growth Factor Basement Membrane Extract (Matrigel)**	R&D Systems<br>Minneapolis, MN, USA	3533-010-02
**DAPI**	BioLegend<br>San Diego, CA, USA	422801
**Dimethyl sulfoxide (DMSO)**	Carl Roth<br>Karlsruhe, Germany	A994.1
**DMEM/F12 Comfort Medium[1]**	Anprotec	AC-LM-0410
**DRAQ5**	BD Biosciences<br>Heidelberg, Germany	564902
**Fetal Constance (FCS)**	Anprotec	AC-SM-0190
**FGF-10 (Recombinant Human)**	Thermo Fisher Scientific	100-26
**FGF-7 (Recombinant Human KGF)**	Thermo Fisher Scientific	100-19
**Formaldehyde solution**	Sigma-Aldrich	252549

**GlutaMAX Supplement**	Thermo Fisher Scientific	35050061
**HEPES solution**	Sigma-Aldrich	H0887
**Isopropanol (2-Propanol)**	VWR<br>Fontenay-sous-Bois, France	20.922.320
**Liquid nitrogen**	Linde<br>Gablingen, Germany	2200919
**N-Acetyl-L-cysteine**	Sigma-Aldrich	A9165-5G
**NC-27 Supplement[2]**	Anprotec	AC-AP-0021
**Nicotinamide**	Sigma-Aldrich	N0636
**Noggin**	Thermo Fisher Scientific	120-10C
**Penicillin-Streptomycin Solution 100X**	Anprotec	AC-AB-0024
**Phosphate-buffered saline (PBS)**	Sigma-Aldrich	D8537
**Primocin**	Invivogen<br>San Diego, CA, USA	ant-pm-05
**Propidium Iodide**	BD Biosciences	556463
**Red blood cell lysis buffer**	Roche Diagnostics<br>Mannheim, Germany	11814389001
**R-Spondin-1**	Thermo Fisher Scientific	120-38
**SB 202190**	Tocris Biosciences	1264/10
**Triton X-100**	Sigma-Aldrich	X100
**TrypLE Express**	Thermo Fisher Scientific	10043382
**Y-27632 dihydrochloride**	TargetMol<br>Boston, MA, USA	TGM-T1725-10MG

[1] Alternatively, Advanced DMEM/F-12 (Thermo Fisher Scientific, Cat. No. 12634010) can be used.

[2] Alternatively, B-27 Supplement (Thermo Fisher Scientific, Cat. No. 17504044) can be used.

Table 4 - Consumables

Item[1]	Company<br>Address	Cat. No.
**1 mL syringe**	BD Biosciences	303172
**10 mL pipette**	Sarstedt<br>Nümbrecht, Germany	861.254.001
**12-well cell culture plate, suspension**	Sarstedt	83.3921.500
**15 mL Falcon tube**	Greiner Bio-One<br>Frickenhausen, Germany	188271
**24-well cell culture plate, suspension**	Sarstedt	83.3922.500
**5 mL pipette**	Sarstedt	861.253.001
**96-well plate**	Thermo Fisher Scientific	442404
**Cryotubes 1.6 mL**	Sarstedt	72.380
**Low Retention Microcentrifuge tubes**	Thermo Fisher Scientific	3451
**Low Retention tips**	Sarstedt	10 µL: 70.3010.275
		20 µL: 70.3020.210
		100 µL: 70.3030.275
		1000 µL: 70.3060.275
**Pasteur pipettes[2]**	VWR	612-1799
**Pre-separation filters (30 µm)**	Miltenyi Biotec<br>Bergisch Gladbach, Germany	130-041-407
**TC insert, for 24-well plates, PET, translucent, pore size: 0.4 µm**	Sarstedt	83.3932.041
**Tissue culture dish (60 x 15 mm)**	Sarstedt	83.3901
**TruLive 3D Dishes**	Luxendo<br>Heidelberg, Germany	80-00310000
**Puradisc 25 syringe filters (0.2 µm)**	Sigma-Aldrich	6780-2502

[1] Consumables provided here are examples and may be replaced by appropriate alternatives from other sources.

[2] Flamed tips prepared in-house.

Table 5 *- Equipment*

Item[1]	Company<br>Address
**CellDrop FL Automated Cell Counter**	DeNovix<br>Wilmington, DE, USA
**Heracell VIOS 250i CO_2 Incubator**	Thermo Fisher Scientific
**Incubation shaker (IS-OS 20)**	Phoenix Instrument<br>Garbsen, Germany
**Infinite 200 Pro Microplate Reader**	Tecan Austria<br>Grödig, Austria
**Multi-Rotator (PTR-60)**	Grant Instruments<br>Royston, UK
**Sunflower Mini-Shaker (PS-3D)**	Grant Instruments
**Water bath (Typ 1008)**	GFL<br>Burgwedel, Germany

[1] Equipment provided here are examples and may be replaced by appropriate alternatives from other sources.
